# Mechanoresponsive lipid-protein nanoglobules facilitate reversible fibre formation in velvet worm slime

**DOI:** 10.1038/s41467-017-01142-x

**Published:** 2017-10-17

**Authors:** Alexander Baer, Stephan Schmidt, Sebastian Haensch, Michaela Eder, Georg Mayer, Matthew J. Harrington

**Affiliations:** 1 Department of Zoology, Institute of Biology, University of Kassel, Heinrich-Plett-Str. 40, 34132 Kassel, Germany; 2 Institute of Organic and Macromolecular Chemistry, Heinrich-Heine-Universität Düsseldorf, Universitätsstraße 1, 40225 Düsseldorf, Germany; 3 Center for Advanced Imaging (CAi), Heinrich-Heine-Universität Düsseldorf, Universitätsstraße 1, 40225 Düsseldorf, Germany; 4 Dept. of Biomaterials, Max Planck Institute of Colloids and Interfaces, Research Campus Golm, 14424 Potsdam, Germany; 5 Dept. of Chemistry, McGill University, 801 Sherbrooke Street West, Montreal, QC H3A 0B8 Canada

## Abstract

Velvet worms eject a fluid capture slime that can be mechanically drawn into stiff biopolymeric fibres. Remarkably, these fibres can be dissolved by extended exposure to water, and new regenerated fibres can be drawn from the dissolved fibre solution—indicating a fully recyclable process. Here, we perform a multiscale structural and compositional investigation of this reversible fabrication process with the velvet worm *Euperipatoides rowelli*, revealing that biopolymeric fibre assembly is facilitated via mono-disperse lipid-protein nanoglobules. Shear forces cause nanoglobules to self-assemble into nano- and microfibrils, which can be drawn into macroscopic fibres with a protein-enriched core and lipid-rich coating. Fibre dissolution in water leads to re-formation of nanoglobules, suggesting that this dynamic supramolecular assembly of mechanoresponsive protein-building blocks is mediated by reversible non-covalent interactions. These findings offer important mechanistic insights into the role of mechanochemical processes in bio-fibre formation, providing potential avenues for sustainable material fabrication processes.

## Abstract (web summary)

Velvet worms expel a fluid slime that, under shear force, forms stiff fibres that can be dissolved and then regenerated. Here, the authors reveal that the recyclability of these biopolymers relies on mechanoresponsive lipid-protein nanoglobules in the slime that reversibly self-assemble into fibrils.

## Introduction

**Fig. 1:**
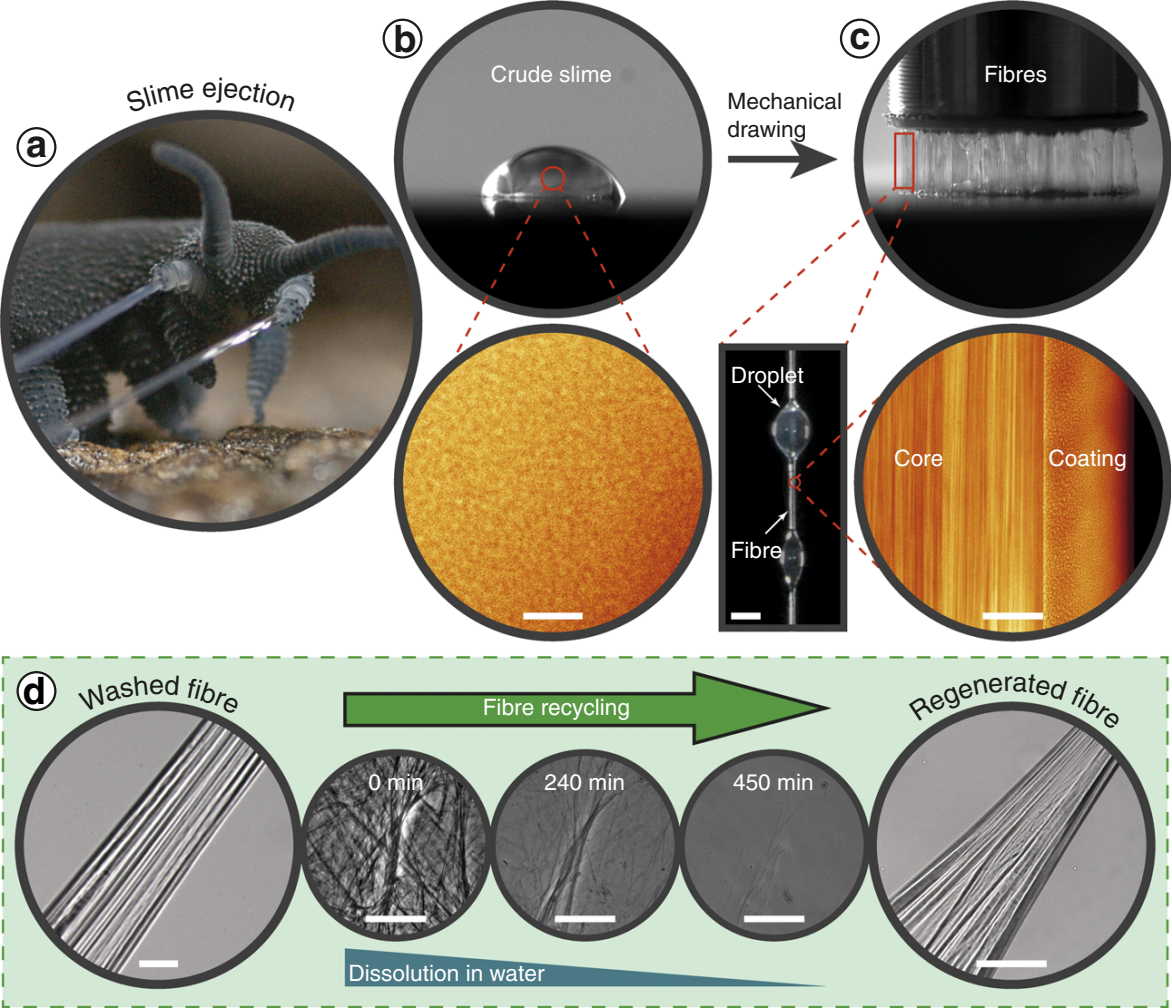
Slime fibres of *Euperipatoides rowelli*. **a** Specimen ejecting capture slime. **b** Crude slime exhibits a particulate texture with confocal microscopy (*scale bar* = 5 µm). **c** Slime forms fibres with sticky droplets via mechanical drawing (inset *scale bar* = 1 mm). Confocal microscopy reveals a fibrous core region, surrounded by a particulate coating (*scale bar* = 15 µm). **d** Water treatment washes away the fibre coating and dissolves the whole fibre on longer time scales. However, dissolved fibre solutions can regenerate new fibres (all *scale bars* = 50 µm)

Organisms that externally extrude structurally and mechanically complex proteinaceous fibres under ambient processing conditions (e.g. spiders, mussels) have emerged as important role models for inspiring sustainable production of advanced polymeric materials^1–4^. Velvet worms (Onychophora) are terrestrial invertebrates inhabiting humid microhabitats of tropical and temperate forests in the southern hemisphere^5^, which deploy a fibre-forming projectile slime for defence and entangling prey^6–8^. The adhesive slime, which is stored as a fluid within slime gland reservoirs, is ejected through papillae—small nozzles on either side of the head—leading to the rapid self-assembly into sticky entangling threads outside the body, which dry and harden into stiff polymeric fibres (Fig. 1a, Supplementary Movie 1)^6, 9–12^. Recent biochemical studies performed on two onychophoran species from the genus *Euperipatoides* indicate that their capture slime is composed primarily of large unstructured proteins and several fatty acid variants, with smaller amounts of protein-bound carbohydrates and free amino acids^11–13^ The ability to easily form fibres outside the body from extracted slime^11, 12^ clearly indicates that the mechanism for fibre assembly must be encoded in these biomolecular building blocks, independent of biological input.

Although there have been a number of investigations over the last century attempting to untangle the mechanisms of slime fibre formation (summarized in Supplementary Table 1)^11–19^, there is still only a very superficial understanding of the molecular mechanisms underlying this fascinating fabrication process. This is further complicated by the fact that these investigations have been performed on a broad range of different onychophoran species. In particular, morphological and physiological differences are evident between the major onychophoran subgroups, the Peripatidae and Peripatopsidae, and it seems likely that there may also be considerable biochemical and structural differences at the level of the slime. Nonetheless, a coherent picture is emerging of a dynamic assembly process in which mechanical and chemical factors contribute to the rapid fluid-to-fibre transition^11, 12, 15–17^.

In the present study, we attempt to reconcile recent biochemical analyses of *Euperipatoides* slime^11, 12^ with earlier studies on the slime formation process from across the two major onychophoran subgroups with a particular focus on the integral role of proteins and lipids^12^. By utilizing a complementary combination of analytical techniques spanning from the molecular to macroscopic level, we gain important mechanistic insights into the dynamic process underlying fibre self-assembly in *Euperipatoides rowelli*.

## Results

### Fibre formation and recycling

Slime manually collected from the velvet worm species *Euperipatoides rowelli* is fluid-like at rest, but when agitated, forms sticky fibres that cure and stiffen through drying, losing their adhesiveness through the process (Fig. 1b, c) as previously reported for other species^17, 18^. Although fibre formation has been reported in many studies, mechanical properties of slime fibres were never previously performed. Tensile mechanical analysis of dried fibres formed under precisely controlled drawing conditions revealed a material stiffness of 4.4 ± 1.2 GPa, comparable to silkworm silk and Nylon®^20^, and a maximum stress and strain of 101.9 ± 20.1 MPa and 3.5 ± 1.2%, respectively (*n* = 18; Supplementary Fig. 1). Confocal microscopic investigation of *E. rowelli* slime reveals the presence of sub-micron globular particles (Fig. 1b), whereas drawn fibres are morphologically divided into a fibrous core sheathed by a coating exhibiting a globular texture resembling the crude slime (Fig. 1c). The coating is continuous with regularly spaced droplets (Fig. 1c)^9^, from which further small fibres could be drawn. Washing the fibre briefly in distilled water removed the coating and droplets, leaving the fibrous core (Fig. 1d). Although previous studies reported that fibres were insoluble in various chemical denaturants^11^, we observed complete dissolution of fibres in distilled water after ~8 h (Fig. 1d, Supplementary Movie 2). The extended time period for full dissociation and the likely dependence of this time on thread thickness may explain why this was not previously observed. Remarkably, new fibres could be regenerated from concentrated dissolved fibre solutions (Fig. 1d), indicating that the biomolecular building blocks are recyclable with water as a solvent. While this is the first demonstration that onychophoran slime fibres can be dissolved and later regenerated, recovery of the native properties from dried slime via rehydration was already reported by Manton & Heatley in 1933 for the *Peripatopsis* species^18^. Whether or not the water-based resolubilization of the fibres has a biological function is presently unclear; however, it might facilitate more rapid digestion of the fibre protein by the organism as previously reported^6^.

### Mechanoresponsive nanoglobules

**Fig. 2:**
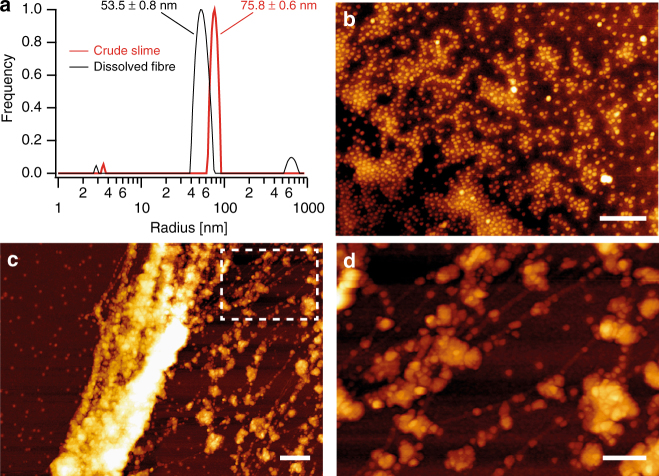
Nanoglobular structure of slime of *Euperipatoides rowelli*. **a** DLS measurements indicating monodisperse nanoscopic particles in crude slime and dissolved fibre solutions. **b** AFM imaging of a 1:100 diluted slime solution physisorbed on a mica surface (*scale bar* = 1 µm). **c** AFM image of 1:100 diluted slime shaken before physisorption on mica (*scale bar* = 2 µm). **d** Zoomed area indicated by dashed box in **c** (*scale bar* = 1 µm)

This extraordinary reversible fibre formation/regeneration process likely stems from an intrinsic dynamic behaviour of the particles comprising the onychophoran slime. To investigate the nanoscale mechanisms underlying fibre formation, we utilized dynamic light scattering (DLS) and atomic force microscopy (AFM). Consistent with confocal microscopy (Fig. 1b), DLS of 20-fold diluted slime indicates the presence of monodisperse aggregates with a hydrodynamic radius of 75.8 ± 0.6 nm (Fig. 2a, Supplementary Fig. 2), further supported by Cryo-TEM measurements (Supplementary Fig. 3), and a less prominent species of 3.5 ± 0.1 nm. Likewise, AFM imaging of 1:100 diluted slime physisorbed and dried onto plasma cleaned silicon surfaces revealed highly uniform nanoparticles with a flattened shape (~100 nm wide and ~10 nm in height), which is likely the result of drying and de-swelling in the lateral directions (Fig. 2b, Supplementary Fig. 4, Supplementary Discussion). Collectively, these measurements suggest the slime constitutes a suspension of spherical monodisperse nanoscopic globules. In fact, the presence of micelles in fluid slime and fresh drawn fibres was previously reported for other species^17, 19^, suggesting that this might be a common feature of onychophoran slime. Since mechanical agitation of slime was found to initiate fibre formation, we also imaged physisorbed, vortexed crude slime samples (1:100 dilution) with AFM, revealing the aggregation of nanoglobules into chains and micron-scale assemblies with nanofibrillar structure (Fig. 2b–d, Supplementary Fig. 4). Furthermore, DLS of dissolved fibre solutions exhibited a similar aggregate size distribution to that of native slime (Fig. 2a), supporting a biomolecular origin of fibre recycling and regeneration and indicating that the disassembled biomolecules spontaneously reorganize into nanoglobules.

### Biomolecular composition of nanoglobules and fibres

**Fig. 3:**
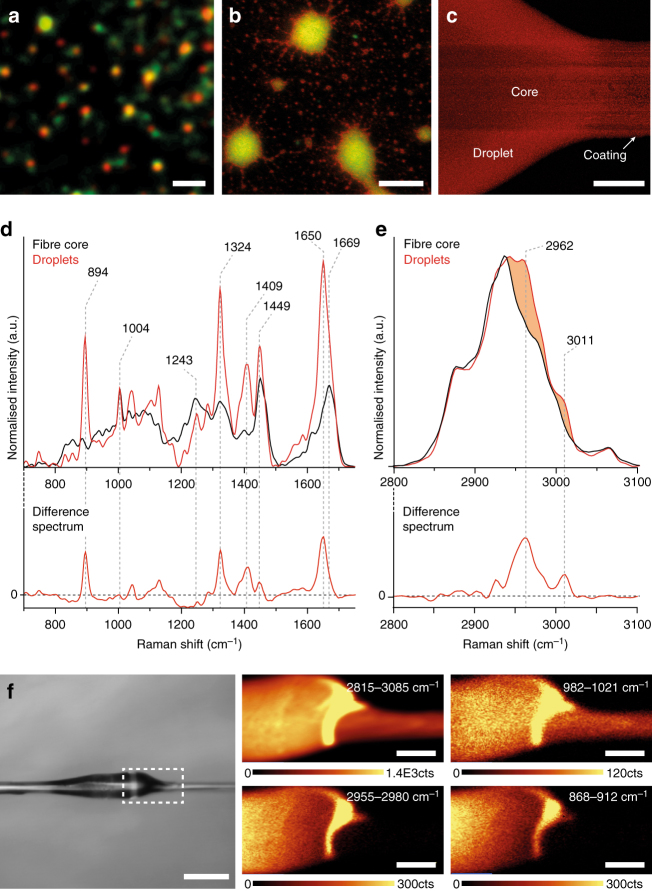
Compositional analysis of slime fibres of *Euperipatoides rowelli*. **a** STED microscopy on 1:100 diluted slime solution, indicating co-localization of lipid (*red*) and protein (*green*) within nanoglobules (*scale bar* = 400 nm) and **b** within larger nanoglobule agglomerates (*scale bar* = 2 µm). **c** Confocal microscopy of m-Cling stained fibre and droplet (*scale bar* = 25 µm). **d**, **e** Raman spectra from 700–1750 cm^−1^ (in **d**) and 2800–3100 cm^−1^ (in **e**) of a washed fibre and fibre droplet normalized to Phe (1004 cm^−1^), with difference spectra below (coating minus core). **f** Raman confocal imaging of the fibre/droplet interface indicated in the microscopy image (*scale bar* = 100 µm) showing integrated peak intensity counts (cts) of the CH band (2815–3085 cm^−1^), Phe peak (982–1021 cm^−1^), and lipid peaks (868–912 cm^−1^ and 2955–2980 cm^−1^) (*scale bars* = 20 µm)

Previous compositional studies indicated that slime from *Euperipatoides* spp. consists primarily of large, disordered proteins and several fatty acid variants^11, 12^. To reconcile this with our current observation of nanoglobule-based fibrillation, we performed sub-micron resolution compositional investigation using stimulated emission depletion (STED) microscopy by staining crude slime with a lipophilic probe (m-Cling)^21^ and an isothiocyanate protein stain (Rhodamine b). STED imaging revealed the co-localization of lipid and protein signals in the nanoglobules (Fig. 3a, Supplementary Fig. 5, Supplementary Discussion), as well as in larger agglomerated particles (Fig. 3b); however, aggregates exhibited a thin lipid-enriched layer surrounding the protein. While STED imaging does not allow us to clearly determine how the lipids and proteins are organized within the nanoglobules at the nanometer scale, it does suggest that a supramolecular interaction between the biomolecules likely controls the formation and regeneration of the monodisperse particles. Staining whole fibres with m-Cling revealed a lower lipid-staining intensity in the core compared to the coating and droplets (Fig. 3c), further supporting that the mechanical formation process disrupts the nanoglobules.

Confocal Raman microspectroscopy was employed to further probe the biomolecular composition of the coating (fibre droplets) and core (washed fibres). While core spectra indicate a primarily protein-based composition (Fig. 3d, e, Supplementary Fig. 6, Supplementary Discussion), coating spectra indicate considerable lipid content in addition. Specifically, difference spectra subtracting core from coating reveal peaks at 2960 and 3010 cm^−1^ assigned to CH_3_ asymmetric stretching and =C-H vibrations in lipids and a sharp intense peak at 895 cm^−1^ consistent with chain-end C-C stretching of solid lipids with well-defined order^22, 23^ (Fig. 3d, e). More ambiguous are sharp bands centred at 1649 and 1324 cm^−1^ that could indicate either C = C stretching and CH_2_ twisting vibrations of lipid molecules^24^ or amide I and amide III bands of α-helical proteins^25, 26^, respectively. However, considering that slime proteins were previously reported to be highly unstructured^11^ and because lipid =C-H stretching (3010 cm^−1^) is typically accompanied by a complementary peak around 1650 cm^−1^, we assign these peaks to lipid vibrations^24, 27^ (Supplementary Fig. 6). Utilizing confocal Raman mapping, we collected spectra at the fibre-droplet interface, showing that lipid-specific peaks appear concentrated in the droplet, whereas total organic and protein-specific peaks are more evenly distributed (Fig. 3f). Considering that Raman spectra from crude slime exhibit a mixture of core and droplet peaks (Supplementary Fig. 6), these findings suggest that the fibre formation process leads to the mechanically driven compositional segregation of the core and coating.

### Integrative model of fibre formation and recycling

**Fig. 4:**
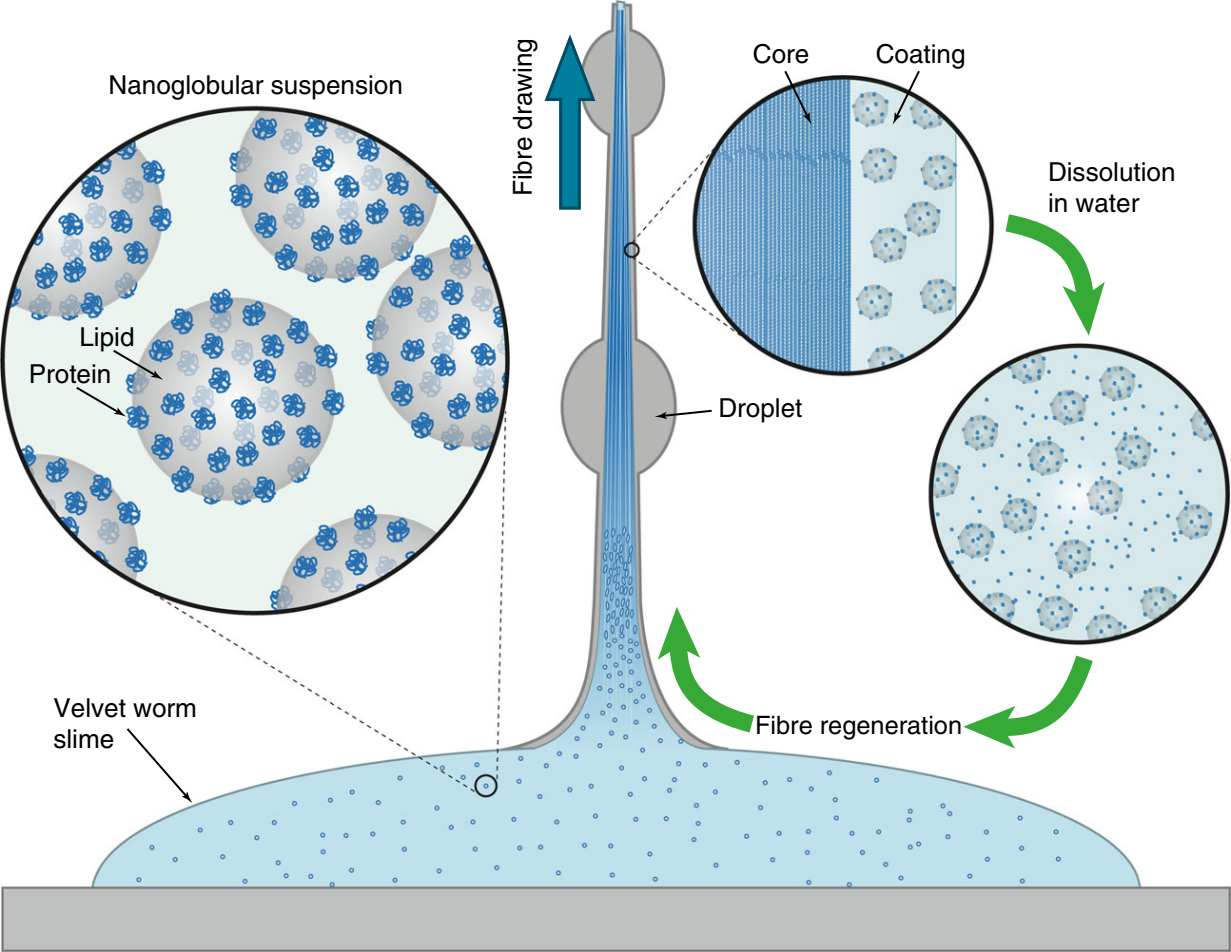
Proposed model of velvet worm slime fibre formation and recycling. The slime of *E. rowelli* consists of monodisperse lipid-protein nanoglobules. Mechanical drawing of fibres produces a protein-rich fibrous core and lipid-enriched coating. However, fibres can dissociate into nanoglobules in water, and can be further regenerated from this solution

There is an urgent need for sustainable approaches to polymer production^28^. Using bio-renewable resources (e.g. cellulose, chitosan) as raw materials for fabrication offers one potential solution^29^. However, much can also be learned by investigating the processes by which biological organisms fabricate polymeric materials in the first place^3, 4^. Our findings indicate that onychophoran slime, in particular that from *E. rowelli*, represents a new paradigm in (bio)-polymeric material assembly, in which stiff fibres can be drawn effortlessly and reversibly from a dense, aqueous suspension of mechanoresponsive lipid-protein nanoglobules (shown schematically in Fig. 4). Biologically speaking, this enables storage of massive amounts of precursor in a small volume, which can be rapidly mobilized, formed into fibres and cured for prey capture. While there are several obvious parallels with the micellar model of spider silk processing^30, 31^, velvet worm fibre processing, in contrast, occurs outside the body and is induced simply by mechanical shear, rather than by subtle transitions in solvent pH and ion content as occurs during silk drawdown^30^. Nanoglobules, thus, appear to be pre-packaged delivery units for biomolecular building blocks that are able to spontaneously self-assemble into fibres upon an external mechanical trigger, which fits well with their biological function as a fast-order projectile capture slime.

Consistent with findings of previous studies of slime from different onychophoran subgroups (Supplementary Table 1), our multi-scale approach clearly indicates that mechanical and chemical factors act synergistically to promote slime fibre formation and regeneration. The presence of monodisperse nanoglobules indicates a supramolecular assembly of biomolecules mediated via lipid-protein interactions^32, 33^, which is disrupted by mechanical agitation, leading to the spontaneous transformation of the nanoglobules into nano- and micron-sized fibrils. Our observations suggest that protein-protein interactions drive fibrillation to form the core, based on the partial segregation of lipids and proteins into the coating and core, respectively. The nature of the interaction between biomolecular building blocks of the onychophoran slime has been a subject of uncertainty in the literature, with some proposing non-covalent interactions^11^ and others suggesting covalent disulfide bonding^14, 16, 17^, although this was never explicitly tested. Our findings clearly indicate that in the case of *E. rowelli* the interactions are non-covalent and water-sensitive, considering that fibres dissolved in water. This is supported by the prominent hydrophobic patches and oppositely charged domains observed in the major slime protein sequences of *E. rowelli* ^12^ and by the previous observation that it is not possible to harden onychophoran slime in seawater^14, 16, 18^, in which abundant ions would have a screening effect on charge-charge interactions. Along these lines, we propose that lipids function to prevent premature protein association and self-assembly until initiated by mechanical shear during slime ejection. It is well documented that lipid interactions can modulate protein structure and assembly^34^; however, further work is required to unravel this aspect of fibre formation. While we are cautious not to over-interpret these results within the larger diversity of onychophoran species, our study provides clear evidence for the synergistic contribution of both mechanical and chemical factors to fibre formation in slime from *E. rowelli*.

Considering that fibre formation is apparently programmed into the biomolecular composition of the slime and entirely dependent on external stimuli, rather than cellular processes, velvet worm slime provides an appealing model for bio-inspired design of sustainably-produced polymeric materials. Indeed, from a polymer production perspective, the fact that fibres with material stiffness on par with Nylon® can be drawn from water under ambient conditions with minimal processing is extraordinary. Furthermore, the observation that fibres dissociate slowly into nanoglobules upon extended exposure to water and that new fibres can be regenerated from this solution has important implications for how humans might fabricate recyclable polymers in the future. One could even envision harnessing a stronger non-covalent interaction such as metal coordination bonding to stabilize the polymer fibres, which would reduce the sensitivity to water, and instead allow recyclability under more defined conditions (e.g. slightly acidic pH), as has been demonstrated with a number of bio-inspired metallopolymeric materials^35^. While there are still many important questions concerning the physicochemical driving forces controlling this process, our study provides important new insights into the assembly mechanism, especially when considered in light of existing literature on onychophoran slime.

## Methods

### Specimens and slime collection

Biomechanical and structural experiments, as well as high speed recordings were performed on the peripatopsid *Euperipatoides rowelli* ^36^. Additional high speed video recordings (Supplementary Movie 1) were performed on the peripatid *Principapillatus hitoyensis* ^37^. Specimens of *E. rowelli* and *P. hitoyensis* were obtained from decaying logs and leaf litter at the corresponding localities and maintained in the laboratory as described previously^10^. The animals were collected and exported under the following permits: (1) the Forestry Commission of New South Wales, Australia (permit no. SL100159); (2) the Gerencia Manejo y Uso Sostenible de RR NN–Ministerio del Ambiente y Energia, Costa Rica (permit numbers 123–2005-SINAC and 014950). All animal treatments complied with the Principles of Laboratory Animal Care and the German Law on the Protection of Animals. Slime samples for each experiment were obtained from 80–100 adult female and male individuals, which were stimulated to eject the slime into 500 µl Eppendorf tubes by carefully touching their anterior ends with a forceps. Collected slime was stored no longer than for 3–4 days at 4 °C to avoid bacterial growth and potential protein degradation.

### Fibre production and mechanical tensile testing

We developed an experimental setup for controlled and reproducible fibre production. A droplet (1 µl) of crude slime was placed on a stainless steel plate, and a stainless steel cylinder (diameter = 1 mm) fixed onto a 5 N load cell was moved with a motor slowly towards the centre of the droplet until it made contact without any compressing force. After a holding time of 1 min, the cylinder with the adhered slime was moved away from the steel plate with a speed of 500 µm/s, drawing slime into a fibre with a length of 13 mm. Fibre samples were allowed to dry until the force reached a constant value (approx. 5 min). The samples were removed afterwards and subjected to tensile testing (*n* = 18). For the tensile tests, the central part of dried fibres (Supplementary Fig. 1) were glued with cyanoacrylate glue (Loctite 454) onto foliar frames. After ~24 h of curing, the samples were fixed onto a custom made tensile tester^38^ and tested in tension with an extension rate of 2 µm/s at a relative humidity of 24 ± 1% and a temperature between 21 and 22 °C. Force was recorded with a 500 mN load cell and strains were measured using video-extensometry, utilizing standardized black markers on the foliar frames. Stresses were calculated based on the cross sections of the fibres, which were determined close to the fracture with an environmental SEM (FEI-ESEM Quanta 600 FEG, 5 keV, 0.75 Torr chamber pressure, LargeField detector). This approach is valid as neither necking nor thinning was observed in dried fibre samples and fracture surfaces were smooth, suggesting brittle fracture. For calculating the tensile stiffness, the slope of the initial linear region of the stress strain curve following sample alignment was determined.

### Confocal laser scanning microscopy (CLSM)

Confocal microscopic imaging was performed on both the crude slime and drawn fibres. Fibres were drawn from a slime solution, which was stained beforehand by adding Atto647N protein dye (Atto-Tec, Siegen, Germany) to the slime sample with a final concentration of 0.6 mM. After drying they were analysed using a confocal laser scanning microscope Leica TCS STED (Leica Microsystems, Wetzlar, Germany). Confocal image stacks were processed using Leica AS AF v2.3.5 (Leica Microsystems).

### Dynamic light scattering (DLS) on diluted slime samples

DLS measurements were conducted with a solution of 50 µl of crude slime in 950 µl water at 20 °C in disposable cuvettes on a Malvern HTTPS Particle Sizer at a fixed scattering angle of 173 degrees. DLS correlation decays and diffusion coefficients of the slime particles were obtain by CONTIN analysis. The Stokes-Einstein relation gave the particle sizes using the viscosities of the diluted slime solution calculated as follows^39^:

1 $$\nu _{{\rm{mix}}}^{{\rm{1/3}}} = {\chi _{{\rm{slime}}}} \, \nu _{{\rm{slime}}}^{{\rm{1/3}}} + {\chi _{{\rm{water}}}} \, \nu _{{\rm{water}}}^{1{\rm{/3}}},$$

where *χ*_slime_ is the mass fraction of slime and *χ*_water_ is the mass fraction of water as diluent, *ν*_slime_ and *ν*_water_ the respective viscosities. With *χ*_water_ = 0.95, *χ*_slime_ = 0.05 and *ν*_slime_ = 119 cp, *ν*_water_ = 0.965 cp we obtain *ν*_mix_ = 1.66 cp.

Average complex viscosity of crude onychophoran slime (*n* = 6) was determined by constant oscillation at very low stress (0.1% strain and 1 Hz) using an oscillation rheometer with cone/plate geometry, Anton Paar MCR 301 (Graz, Austria).

### Atomic force microscopy (AFM) and imaging

AFM imaging was conducted in tapping mode on a Nanowizard III (JPK Instruments AG, Berlin) using standard tapping mode cantilevers with a nominal spring constant of 40 N/m (µMash, Bulgaria). Surfaces of physisorbed nanoglobules were obtained by first diluting the slime with ultrapure water (1:100), then casting a droplet on RCA-cleaned glass (H_2_0_2_, NH_3_, water, 1:1:5, at 70 °C for 30 min), followed by immediate rinsing with water and drying under nitrogen stream. Slime fibres were prepared by casting vortexed (40 s) slime solution (1:100 dilution) on RCA cleaned glass.

### Cryogenic transmission electron microscopy (Cryo-TEM)

Diluted slime samples (1:2 dilution with water) were prepared for Cryo-TEM analysis using a plunge freezer (FEI Vitrobot™, Hillsboro, Oregon, USA) with liquid nitrogen and ethane and were studied with a 200 keV JEOL JEM 2100 transmission electron microscope (Peabody, MA, USA).

### Lipid and protein staining using STED

Crude slime was gently diluted (1:100) in 10 mM phosphate buffer (pH 4.9) to a final volume of 100 µl. Proteins were labelled nonspecifically by addition of Rhodamine B isothiocyanate (RITC, Sigma Aldrich, Germany) at a concentration of 4 µM. Lipid labelling was carried out by addition of the membrane marking dye mCling (mCling-Atto647N-labeled, Synaptic Systems, Germany) at a final concentration of 400 nM. The diluted slime was incubated with the dyes at room temperature for 10 min (mCling) or 1 h (co-staining with RITC), respectively and transferred to charged or PEGylated glass chambers. Settling and spreading of the slime was allowed for another 15 min before microscopic and nanoscopic measurements were applied. Confocal and STED measurements were performed using a TCS SP8 STED 3X (Leica) equipped with an HC PL APO CS2 100x objective (NA 1.4) at a scan speed of 1000 Hz. As excitation laser wavelengths, 649 nm for mCling-Atto647N and 540 nm for RITC were chosen for both techniques. As depletion lasers for STED measurements, 660 nm in case of RITC and 775 nm in case of mCLing-Atto647N were used. Detection range for the emitted fluorescent signals in confocal microscopy and STED was set from 547 to 600 nm for RITC and from 660 to 765 nm for mCling-Atto647N. In co-staining experiments, detection of the different dyes was carried out in a sequential measurement setup. Finally, deconvoluted data of STED measurements were calculated using standard algorithms of Huygens software (Huygens professional, Scientific Volume Imaging, Netherlands).

### Confocal Raman microspectroscopy

Crude and washed slime fibres were fixed in the dried state onto metal frames for Raman spectroscopic measurements. Individual Raman spectra were collected from droplets and washed core regions with a confocal Raman microscope (alpha300; WITec) equipped with a piezoelectric scan stage (P-500, Physik Instrumente), and a Nikon objective (20X, NA = 0.40). A laser (λ = 532 nm) was focused onto the sample and Raman scattering was detected with a CCD camera (DV401-BV; Andor) behind a spectrometer (UHTS 300; WITec) with a spectral resolution of 3 cm^−1^. Reported spectra for fibre droplets and washed fibres are each averages of at least six different spectra acquired using an integration time of 0.5 s with 30 integrations. At least five different crude and washed fibres were measured with similar results. Using the OPUS software (version 7.0, Bruker Optik), each spectra was background corrected prior to averaging and their spectral intensities were normalized to the Phe peak at 1004 cm^−1^. Difference spectra were acquired following Phe peak normalization by subtracting the average core spectrum from the average coating spectrum. Mapping of the fibre-droplet interface of a crude fibre was performed using a 0.5 μm step size with an integration time of 0.1 s per step. Raman mapping images were generated using the filter manager function in the ScanCtrlSpectroscopyPlus software (WITec) by integrating the intensity of the CH stretching band (2815–3085 cm^−1^), the Phe peak of proteins (982–1021 cm^−1^), as well as the CH_3_ asymmetric stretching (2955–2980 cm^−1^) and chain-end C-C stretching (868–912 cm^−1^) bands of lipids, respectively.

### Data availability

The data supporting the findings of this study are available from the authors upon reasonable request.

## Electronic supplementary material

Supplementary Information

Description of Additional Supplementary Information

Supplementary Movie 1

Supplementary Movie 2
