# Supplementary material for: Mechanoresponsive lipid-protein nanoglobules facilitate reversible fibre formation in velvet worm slime
Source: Nat Commun. 2017 Oct 17;8:974. doi: 10.1038/s41467-017-01142-x (PMC5645397; doi:10.1038/s41467-017-01142-x)
Supplement: Supplementary file 1 — Supplementary Information [file 41467_2017_1142_MOESM1_ESM.pdf]

Supplementary Table 1: Summary of findings of prominent studies dealing with structure, composition and properties of the onychophoran slime.

**Reference**	**Manton & Heatley (1937)**[1]	**Röper (1977)**[2]	**Ruhberg & Storch (1977)**[3]	**Read (1985, unpublished PhD thesis)**[4]	**Monge-Nájera et al. (1993)**[5]
**Species studied**	Peripatopsidae<br>*Peripatopsis moseleyi* and *P. sedgwicki*<br>(South Africa)	Peripatopsidae<br>*Peripatopsis moseleyi*<br>(South Africa)	Peripatopsidae<br>*Peripatopsis moseleyi*<br>(South Africa)	Peripatidae<br>*Macroperipatus torquatus*<br>(Trinidad)	Peripatidae<br>*Epiperipatus biolleyi*<br>(Costa Rica)
**Composition**	proteins of low molecular weight (LMW)	no intra-individual differences;<br>84% water;<br>16 % protein;<br>free amino acids (Gly, Glu)			
**Properties**	pipettable fluid;<br>pH value 5.2–5.4;<br>opalescence pH value dependent;<br>bitter taste, without smell or colour;<br>fresh fibres sticky;<br>dried fibres non-sticky, brittle;<br>dried slime in fresh water adhesive before solubilisation	sticky fluid forms sticky fibres;<br>dries in air with loss of adhesiveness	fibre-forming secretion		slime dries in air in 3s;<br>soluble in sea water (no fibre formation, non-adhesive);<br>fibre formation in fresh water;<br>sticky for 20 h in fresh water
**Structure**		elastic fibres possibly due to micellar structure	fresh fibres micellar;<br>dried fibres non-micellar;<br>irregular surface of cross section		
**Slime/fibre curing**		possibly cross-linking via disulfide bridges		mechanical shock possibly causes polymerisation	suggest a chemical hardening mechanism

Supplementary Table 1 (continued).

**Reference**	**Mora et al. (1996)[6]**	**Benkendorff et al. (1999)[7]**	**Haritos et al. (2010)[8]**	**Baer et al. (2014)[9]**	**Present study**
**Species studied**	Peripatidae<br>*Epiperipatus* sp.<br>(Costa Rica)	Peripatopsidae<br>*Euperipatoides kanangrensis*<br>(Australia)	Peripatopsidae<br>*Euperipatoides rowelli*<br>(Australia)	Peripatopsidae, 10 species<br>Peripatidae, 2 species<br>(Tasmania, mainland Australia, Costa Rica, Brazil)	Peripatopsidae<br>*Euperipatoides rowelli*<br>(Australia)
**Composition**	HMW protein bands conserved within Peripatidae;<br>LMW protein bands species-specific	~90% water;<br>protein (LMW and HMW, partly O- or N-glycosylated);<br>sugar, lipids and nonylphenol	three categories of proteins;<br>majority of proteins of high molecular weight (HMW) and proline-rich;<br>very low cysteine content	proteins of different size;<br>intra-specific conservation;<br>HMW proteins conserved within major subgroups;<br>LMW proteins species-specific	proteins and lipids
**Properties**	slower hardening or fibres in freshwater<br>slime soluble in sea water, SDS and β-Mercaptoethanol	slime forms fibres of high tensile strength<br>droplets of sticky fluid along fibres	slime forms fibres	pipettable;<br>soluble in distilled water, SDS and β-mercaptoethanol	low viscosity of slime;<br>fibre formation due to mechanical impact;<br>fibre stiffness like in silkworms and Nylon®;<br>fibres soluble in distilled water
**Structure**		amino acid composition suggests collagen-like structure	proteins lack ordered secondary structures		proteins and lipids exist as ~70 nm monodisperse nanoglobules<br>fibres consist of protein-enriched core and lipid-rich coating<br>re-formation of nanoglobules after resolubilization
**Slime/fibre curing**	possible existence of disulfide bridges (not tested)		glass transition due to evaporative water loss;<br>non-covalent cross-linking of charged and hydrophobic regions of proteins		reversible self-assembly of nanoglobules into fibres due to mechanical impact;<br>water-sensitive, non-covalent interactions of proteins

Quasi-static tensile mechanical testing of velvet worm slime fibres

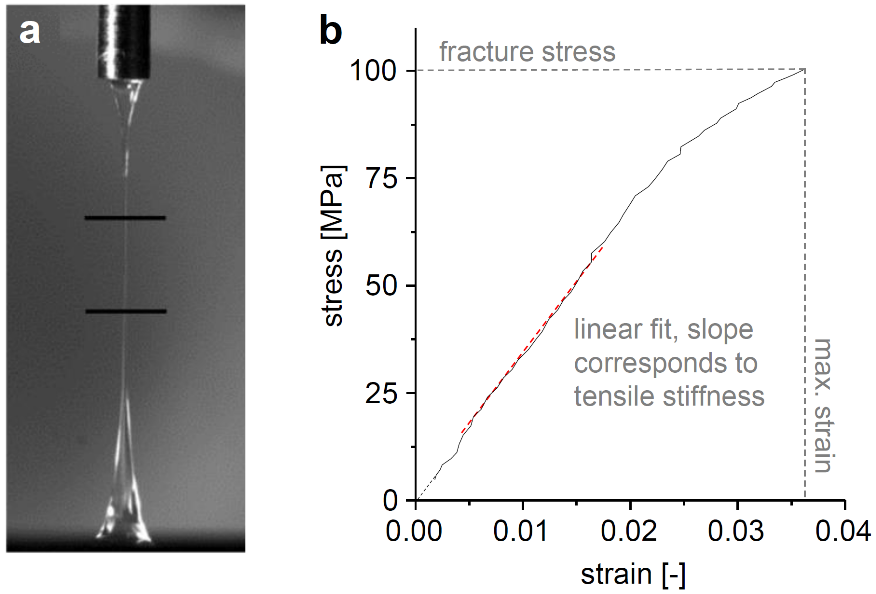

	mean value (n=18)	standard deviation
tensile stiffness	4386 MPa	1145 MPa
breaking stress	101.9 MPa	20.1 MPa
max. strain	3.5 %	1.2 %
cross sectional area	859 μm²	430 μm²

Supplementary Figure 1. Mechanical testing of slime fibres from *E. rowelli*. A) Fibres were drawn from velvet worm slime by pressing a metal cylinder (diameter = 1 mm) into 1 μl of slime and pulling it away at a speed of 500 μm/s, allowing the formation of highly uniform and reproducible fibres. Black horizontal lines indicate approximately the regions where the fibres were fixed to the sample holders for tensile testing. B) Typical stress-strain curve of a dried fibre stretched at an extension rate of 2 μm/s. The initial phase of the stress-strain curve, assigned to alignment processes has been removed and replaced by the dashed line.

Supplementary Discussion

Dynamic light scattering (DLS) experiments on diluted slime samples:

DLS measurements were conducted using crude slime, as well as regenerated slime that was prepared by dissolving a dried slime fibre in distilled water. The crude slime sample was analysed to measure the hydrodynamic radii of the nanoglobular particles in the slime and to confirm their high monodispersity. DLS analysis gave an overall size of 75.8 ± 0.6 nm and a DLS polydispersity index <0.1 indicating highly monodisperse particles (Supplementary Fig. 2). The second smaller peak observed at 2–4 nm may indicate the presence of freely dissolved proteins.

The dissolved fibre sample was analysed to investigate the reversibility of nanoglobule formation in water. Here, the fibre was allowed to dissolve in water overnight at room temperature. DLS results confirm the presence of monodisperse particles after dissolution with a size distribution similar to particles of the crude slime (Supplementary Fig. 2). In addition, a larger peak is observed at >500 nm, which may indicate that equilibrium was not quite achieved within dissolution time, i.e. incomplete nanoglobule formation. The main peak also indicates that the nanoglobular particles in the dissolved fibre solution are slightly smaller (50–60 nm) and possess a slightly larger polydispersity index of >0.15 compared to the crude slime.

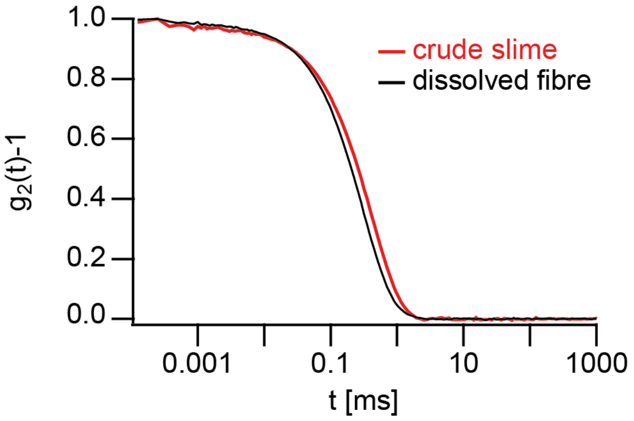

Supplementary Figure 2. DLS measurements on slime solutions *from E. rowelli*. DLS correlation functions for crude slime (red curve) and regenerated slime from the dissolution of a slime fibre in water overnight (black curve).

Cryo-transmission electron microscopy of onychophoran slime

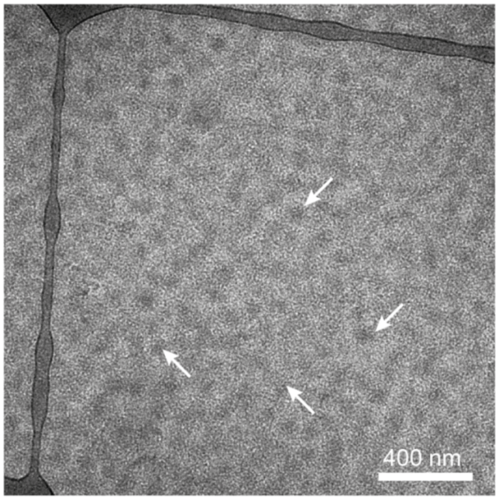

Supplementary Figure 3. Cyro-transmission electron microscopy (TEM) of crude slime from *E. rowelli*. Cryo-TEM micrograph of 1:2 diluted onychophoran slime indicates the presence of spherical nanoglobules of approximately 70 nm.

Atomic force microscopy analysis of nanoglobular and fibre nanostructure

The structure of dispersed slime components and cured slime fibres was analysed on the nanoscale by AFM. Monodisperse nanoscopic objects were found when a diluted slime droplet was allowed to rest on the surface followed by rinsing and drying (Supplementary Fig. 4a). The particles have a flat structure (width ~100 nm, height ~10 nm), which could be caused by drying and de-swelling in the vertical direction, whereas lateral de-swelling was hindered due to adhesion. This behaviour is typical for soft, swollen objects like microgels and protein particles[10]. Therefore, the root mean square roughness (RMS roughness) of these surfaces is small, about 5 nm. Nanoscopic objects were not observed on the surface of completely dried slime droplets (Supplementary Fig. 4b). There are no visible phase boundaries and the surface shows a very low roughness, RMS ~2.5 nm. Clear phase boundaries were also not seen when imaging the cross-section of a slime fibre (Supplementary Fig. 4d) and the roughness was again rather small (RMS 4.5). This indicates that the nanoglobular vesicles fuse in the bulk of the drying slime droplet, thus showing no regular phase boundaries. Bundles of fibres could be observed when the slime was dispensed on a spinning solid support (Supplementary Fig. 4c). In this case, the shear forces induced fibre formation. Interestingly, the fibres consisted of smaller fibrils with diameters similar to the size of the micelles. Fibres were also found when the slime solution was vortexed prior to adsorption on solid support (Fig. 2c-d).

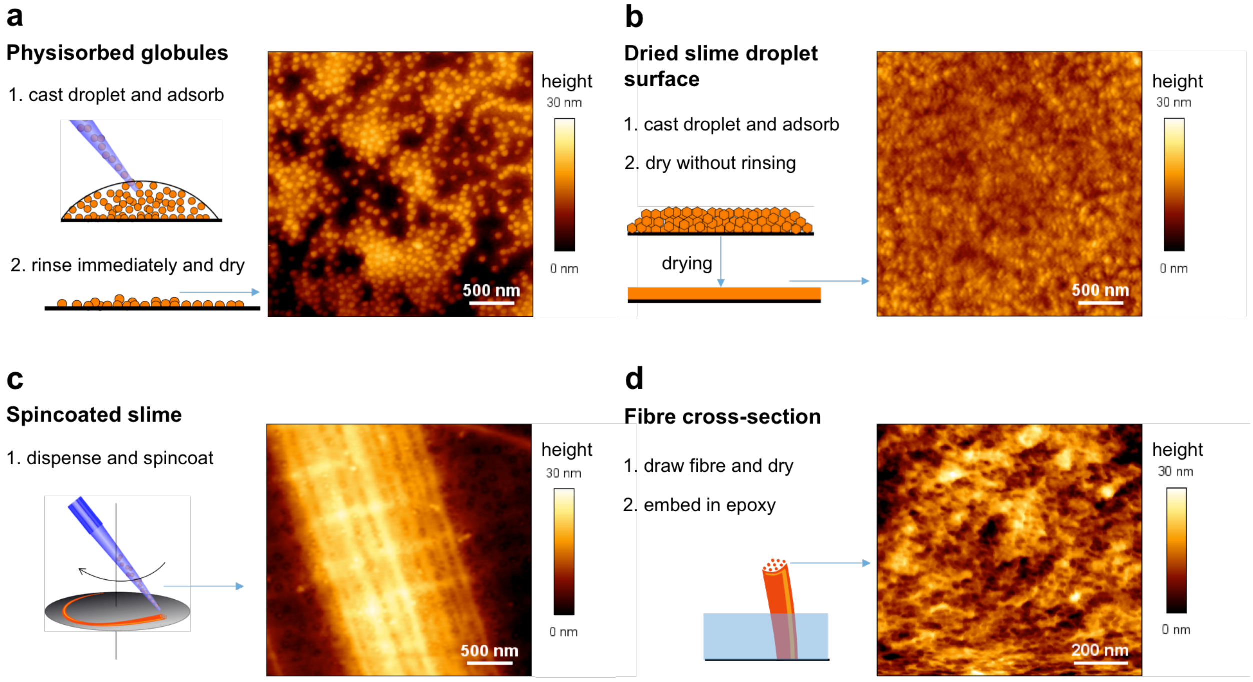

Supplementary Figure 4. AFM studies on slime samples *from E. rowelli*. a) AFM image of physisorbed nanoglobules by immediate rinsing the surface after slime deposition. With this procedure, individual nanoglobules can be observed. b) Surface of a dried slime droplet (no dilution) shows no individual nanoglobules c) Image of a spincoated slime sample (1:10 dilution) shows fibrils forming a larger fibre. d) In the

cross-section of a drawn fibre from crude slime no clear phase boundaries of individual nanoglobules could be identified.

Supplementary Experimental Procedures: AFM imaging was conducted in tapping mode on a Nanowizard III (JPK Instruments AG, Berlin) using standard tapping mode cantilevers with a nominal spring constant of 40 N/m (µMash, Bulgaria). Images of the physisorbed nanoglobules were obtained by first diluting the slime with ultrapure water, then casting a droplet on plasma cleaned glass, followed by immediate rinsing with water and drying under nitrogen stream. The dried slime droplet sample was prepared by simply drying a crude slime droplet in ambient condition. The spincoated sample was prepared from a 1:10 dilution on cleaned glass slide spun at 3000 RPS. The sample for imaging fibre cross-section was prepared by drawing a fibre from crude slime, drying it on a petri dish and embedding the fibre vertically in commercial two-component epoxy resin. After curing the epoxy resin, the fibre was manually broken close to the epoxy surface and then imaged. The vortexed sample was first diluted 1:100 and then vortexed for at least 40 seconds at maximum intensity in a 1.5 ml Eppendorf tube. A droplet of the vortexed solution was then spread on cleaned glass slide, then rinsed with water and dried with nitrogen.

Non-deconvoluted STED images

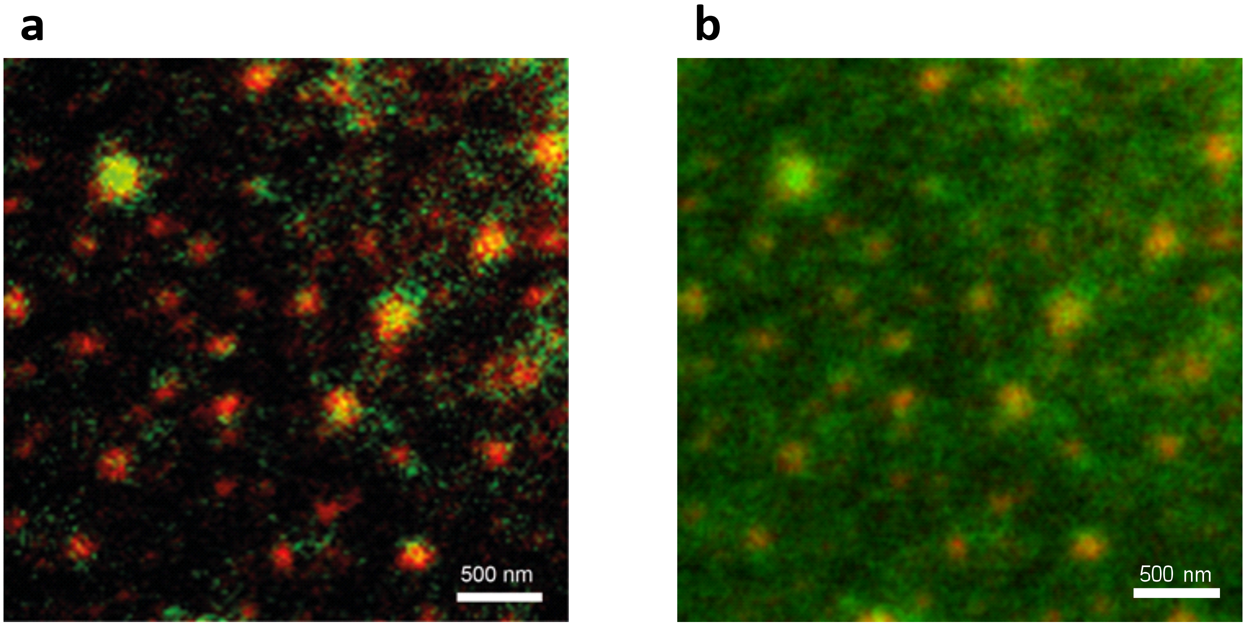

Figure S5. Non-deconvoluted STED images of nanoglobules. a) contrast optimized image; b) unprocessed raw image. The overlay of Rhodamine (green) and m-Cling (red) channel suggests that both channels correspond spatially. This indicates that the nanoglobules are composed of fatty acids, as well as proteins.

STED microscopy provides resolution down to a few tens of nanometer and was used to image single nanoglobules. The main text shows a deconvoluted STED image of the nanoglobules (Fig. 3a). Deconvolution helped to reverse the optical distortion that takes

place due to the diffraction limitation and noise by using a software algorithm provided by the microscope manufacturer (Leica Application Suite, Leica Microsystems Germany). Supplementary Fig. 5a shows the original non-deconvoluted image and Supplementary Fig. 5b shows the completely raw unprocessed image. As seen with the deconvoluted image (Fig. 3a) there is a correlation between the green (rhodamine B) and red (m-Cling) channel. This results in the yellow/orange color of the nanoglobules in the overlay and indicates that the nanoglobules contain fatty acids, as well as proteins. Note that due to thermal drift and diffusion of the nanoglobules both colors do not perfectly match as the channels were imaged consecutively. It is also noteworthy that the m-Cling channel provided better resolution, as evidenced by the more diffuse appearance of the rhodamine label.

Raman spectroscopic analysis of velvet worm slime and fibres

Confocal Raman microspectroscopy provides information about biomolecular composition with submicron resolution and was used in the present study to investigate dried fibre droplets (coating only), dried washed fibres (core only) and dried crude slime. Considering that the slime consists of both lipids and proteins[7], we were cautious in assigning Raman spectral peaks, as many characteristic vibrational bands of lipids and proteins fall within similar spectral ranges[11,12]. Regardless of the above-mentioned caveats, there are major differences between core and coating Raman spectra (Supplementary Fig. 6), indicating real compositional and conformational differences between the two components of slime.

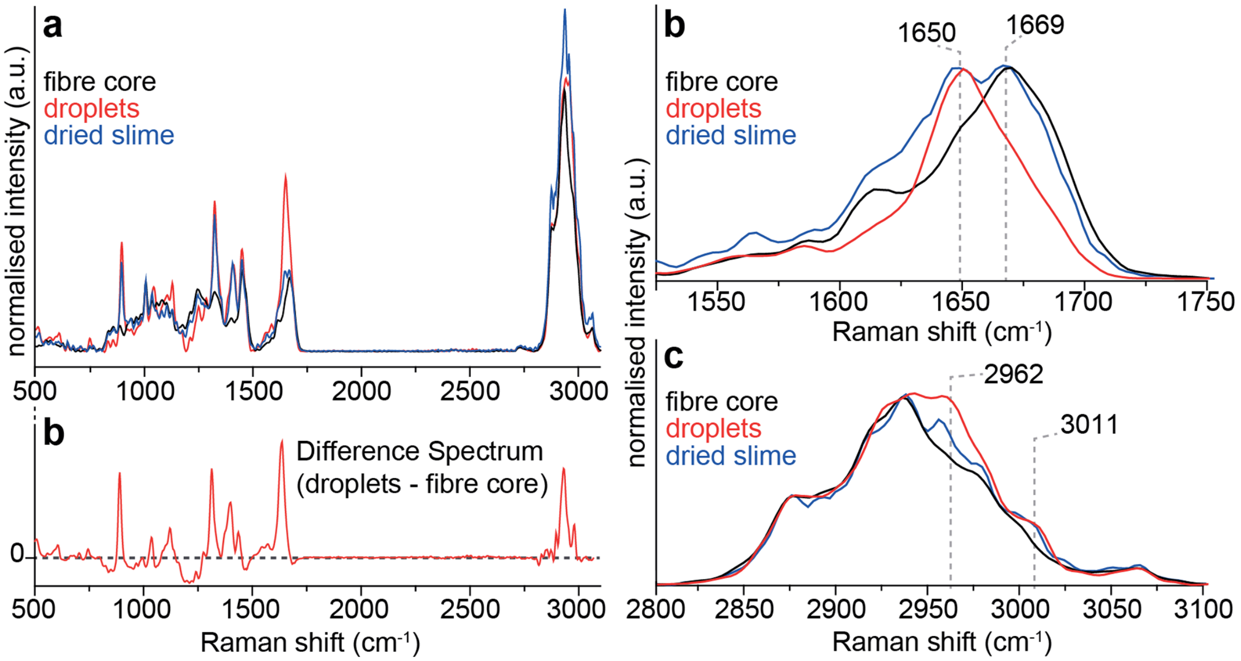

Supplementary Figure 6. Raman spectroscopy of crude slime and fibres from *E. rowelli*. a) Full range of averaged Raman spectra from dried crude slime, washed fibre core and coating droplets normalized to the maximum intensity of the Phe peak at 1004 cm^{-1}. b) Raman difference spectrum subtracting Phe-normalized fibre core spectrum

from droplet spectrum. c) Closeup of amide I region from panel (a) normalized to the maximum intensity in the range of 1525–1750 cm^{-1}. d) Close-up of CH stretching region from panel (a) normalized to the maximum intensity in the range of 2850–3100 cm^{-1}.

Washed fibre spectra are consistent with a primarily protein-based composition based on comparison to other protein-based materials (Supplementary Fig. 6a)[12]. Specifically, peaks in the amide I (1620–1700 cm^{-1}) and amide III (1200–1380 cm^{-1}) bands indicate a disordered or extended protein backbone structure (e.g. beta strand, PPII, random coil), as previously proposed based on FTIR spectroscopy and X-ray diffraction of fibres, as well as from predicted secondary structures of slime protein sequences[8]. Additionally, prominent peaks at 833, 855, 1004 and 1616 cm^{-1} indicate the presence of protein-specific aromatic amino acids (tyrosine and phenylalanine), which are prominent in crude slime proteins (Phe + Tyr = ~7 mol%)[7,8].

Although spectra from the coating droplet also indicate a clear protein component based on similarities with core spectra, there are several prominent coating-specific peaks at positions that are not immediately assignable to protein or amino acid side chain vibrations[12]. In order to highlight the coating-specific peaks, we utilized spectral subtraction by first normalizing averaged spectra from the core and coating to the intensity of the phenylalanine peak at 1004 cm^{-1}, assuming similar proteins are present in both the core and coating (Supplementary Fig. 6b). This is a relatively safe assumption considering that crude slime amino acid composition shows a similar Phe content to the individually isolated slime proteins[7,8]. By subtracting the fibre core spectrum from that of the adhesive, a number of strong and sharp peaks are revealed, many of which do not correspond to protein-based vibrational peaks, but rather are consistent with lipid chain vibrations (Supplementary Fig. 6b,d). Most importantly, strong peaks in the difference spectrum at 2960 cm^{-1} and 3010 cm^{-1} in the CH vibrational band can be confidently assigned to CH_3 asymmetric stretching and =C-H stretching in lipids[11,12] and a sharp intense peak at 895 cm^{-1} is consistent with chain-end C-C stretching of solid lipids[13]. Notably, the intensity of this peak has been previously reported to indicate highly ordered arrangement of the lipid chains in the solid form, but is less prominent in liquid phase lipids[13].

The regions typically assigned to amide I and amide III vibrations also contain strong and sharp peaks in the difference spectrum that would indicate a dominant α-helical protein structure (Supplementary Fig. 6A, C); however, previous investigations of crude fibres (possessing coating and core) did not detect the presence of α-helical structure with XRD or FTIR spectroscopy, nor do any of the protein sequences reported contain any dominant predicted helical structure[8]. Rather, the proline-rich proteins comprising the slime fibres are predicted to be largely unstructured[8]. As an alternative hypothesis, we propose that these peaks arise from the lipid backbone C=C stretching (1650–1675 cm^{-1}) and CH_2 twisting (~1310 cm^{-1})[11]. In support of this assignment, a peak at ~1650 cm^{-1} is almost always observed alongside the peak at ~3010 cm^{-1} in unsaturated lipids, since they both arise from vibrations arising from carbon double bonds in the lipid backbone[11,14].

Supplementary References

1 Manton, S. M. & Heatley, N. Y. Studies on the Onychophora. IV, II The feeding, digestion, excretion and food storage of *Peripatopsis*. Philos. T. Roy. Soc. B **227**, 411–464, (1937).

2 Röper, H. Analytical investigations on the defensive secretions from *Perpatopsis moseleyi* (Onychophora). *Zeitschrift für Naturforschung* **32**, 57–60, (1977).

3 Ruhberg, H. & Storch, V. Über Wehrdrüsen und Wehrsekret von *Peripatopsis moseleyi* (Onychophora). *Zoologischer Anzeiger* **198**, 9–19, (1977).

4 Read, V. M. S. J. *The ecology of Macroperipatus torquatus (Kennel) with special reference to feeding and a taxonomic review* (University College of North Wales, Bangor., Ph.D. Thesis, 1985).

5 Monge-Nájera, J., Barrientos, Z. & Aguilar, F. Behavior of *Epiperipatus biolleyi* (Onychophora: Peripatidae) under laboratory conditions. *Revista de Biología Tropical* **41**, 689–696, (1993).

6 Mora, M., Herrera, A. & León, P. Análisis electroforético de las secreciones adhesivas de onicóforos del género Epiperipatus (Onychophora: Peripatidae). Revista de Biología Tropical **44**, 147–152, (1996).

7 Benkendorff, K., Beardmore, K., Gooley, A. A., Packer, N. H. & Tait, N. N. Characterisation of the slime gland secretion from the peripatus, *Euperipatoides kanangrensis* (Onychophora : Peripatopsidae). *Comp. Bioch. Physiol. B-Biochem. Molec. Biol.***124**, 457-465, (1999).

8 Haritos, V. S. *et al.* Harnessing disorder: onychophorans use highly unstructured proteins, not silks, for prey capture. *P. Roy. Soc. B-Biol. Sci.* **277**, 3255-3263, (2010).

9 Baer, A., de Sena Oliveira, I., Steinhagen, M., Beck-Sickinger, A. G. & Mayer, G. Slime protein profiling: a non-invasive tool for species identification in Onychophora (velvet worms). *J. Zool. Syst. Evol. Res.* **52**, 265–272, (2014).

10 Schmidt, S., Hellweg, T. & von Klitzing, R. Packing Density Control in P(NIPAM-co-AAc) Microgel monolayers: Effect of surface charge, pH, and preparation technique. *Langmuir* **24**, 12595-12602, (2008).

11 Czamara, K. *et al.* Raman spectroscopy of lipids: a review. *J. Raman Spectroscop.* **46**, 4-20, (2015).

12 Movasaghi, Z., Rehman, S. & Rehman, I. U. Raman spectroscopy of biological tissues. *Appl. Spectroscop. Rev.* **42**, 493-541, (2007).

13 Brown, K. G., Bicknellbrown, E. & Ladjadj, M. Raman-active bands sensitive to motion and conformation at the chain termini and backbones of alkanes and lipids. *J. Phys. Chem.* **91**, 3436-3442, (1987).

14 Billecke, N. *et al.* Perilipin 5 mediated lipid droplet remodelling revealed by coherent Raman imaging. *Integr. Biol.* **7**, 467-476, (2015).
