# Supplementary material for: Mechanoresponsive lipid-protein nanoglobules facilitate reversible fibre formation in velvet worm slime
Source: Nat Commun. 2017 Oct 17;8:974. doi: 10.1038/s41467-017-01142-x (PMC5645397; doi:10.1038/s41467-017-01142-x)
Supplement: Supplementary file 2 — Description of Additional Supplementary Information [file 41467_2017_1142_MOESM2_ESM.pdf]

**Description of Additional Supplementary Files**

File Name: Supplementary Movie 1
Description: High speed video recording of slime ejection from the peripatid Principapillatus hitoyensis.

File Name: Supplementary Movie 2
Description: Resolubilzation of onychophoran slime fibers in distilled water. Fibers were placed in distilled water and observed with a light microscope over a period of ~8 hours.
